# Harnessing marine sulfated polysaccharides to inhibit norovirus: from seaweed to solution

**DOI:** 10.1128/spectrum.02445-25

**Published:** 2026-03-09

**Authors:** Barbara C. Wimmer, Joel T. Kidgell, Mark von Itzstein, Thomas Haselhorst, Grant S. Hansman

**Affiliations:** 1 Marinova Pty Ltd 371032 , Cambridge, Tasmania, Australia; 2 Institute for Biomedicine and Glycomics (IBG), Griffith University, Gold Coast Campus, Gold Coast, Queensland, Australia; Cornell University College of Veterinary Medicine, Ithaca, New York, USA

**Keywords:** antiviral, norovirus, structural modeling, histo-blood group antigens

## LETTER

**Fig 1:**
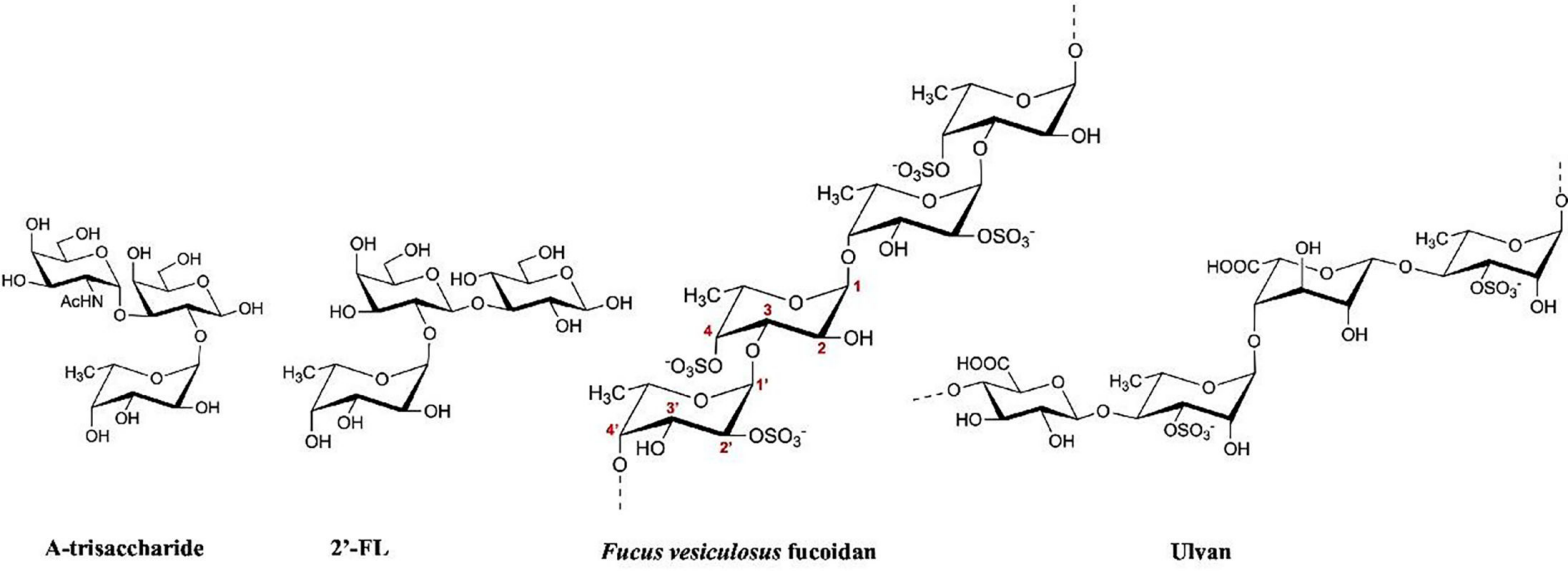
Hayworth projections of an HBGA A-trisaccharide, HMO 2′-FL, and representative sections of *Fucus vesiculosus* fucoidan and ulvan. A-trisaccharide is 6-deoxy-a-L-galactopyranosyl-(1→2)-[2-acetamido-2-deoxy-a-D-galactopyranosyl-(1→3)]-D-galactose (GalNAca1,3(Fuc-a1,2)Gal). The 2′-FL is 6-deoxy-α-L-galactopyranosyl-(1→2)-β-D-galactopyranosyl-(1→4)-D-glucose (Fucα1,2Galβ1,4Glc). The *Fucus vesiculosus* fucoidan (type II) has alternating α-L-fucose-(1, 3)-L-fucose-(1, 4) with sulfate groups and can also contain other monosaccharides like glucose, galactose, and xylose, along with acetyl groups and uronic acids (not shown). Ulvan is a highly complex and variable sulfated hetero polysaccharide that is primarily composed of repeating disaccharide units typically comprising rhamnose-3-sulfate, glucuronic acid, and iduronic acid with lesser amounts of xylose, mannose, arabinose, and galactose. These polysaccharides are generally a few 100 kDa, and have been reported up to 1000 kDa.

Human noroviruses are the leading cause of acute gastroenteritis outbreaks worldwide, responsible for >685 million infections annually (1). Still, there are no approved vaccines, antivirals, or specific treatments. One promising approach involves the use of compounds that block norovirus attachment to histo-blood group antigens (HBGAs), which serve as essential co-factors for viral entry (2, 3). Human milk oligosaccharides (HMOs), such as 2′-fucosyllactose (2′-FL), have already demonstrated this capability, where the primary fucose moiety of HMOs bound precisely at the HBGA pocket on the capsid (4–10). Building on this concept, we further investigated the antiviral potential of polysaccharide (fucose-based) fucoidans and ulvan extracted from various seaweed species (Fig. 1), which have shown promising inhibition capacities for noroviruses (11–14) and other viruses (15).

**Fig 2:**
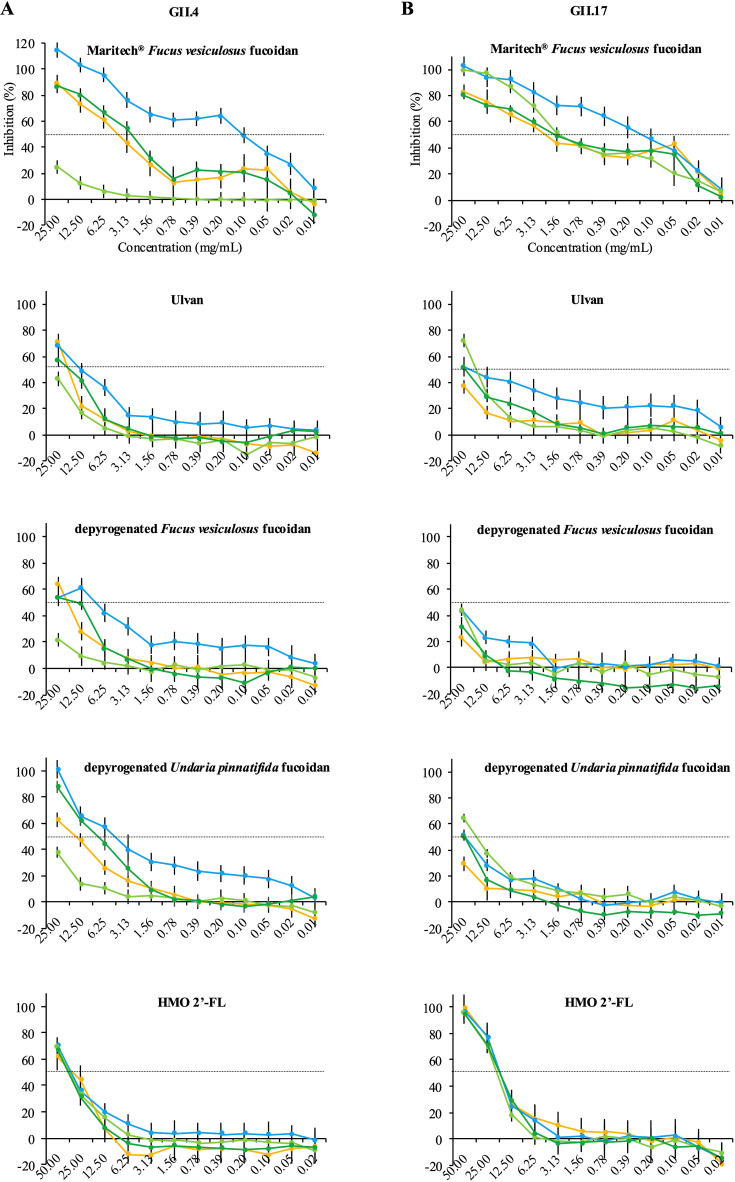
Inhibition profiles of fucoidans, ulvan, and 2′-FL. Blocking assays were performed using a previously described method (4) using saliva samples representing blood types A, B, and O from healthy donor adults. Triplicate wells were coated with a 1:200 dilution of heat-inactivated (99°C, 15 min) human saliva sample overnight at 4°C. Serial dilutions starting at 50 mg/mL of Maritech *Fucus vesiculosus* fucoidan (≥85% fucoidan, FVS2024564), ulvan (≥85%, UOU2022559), depyrogenated *Fucus vesiculosus* fucoidan (≥90%, DSFVF2024520), depyrogenated *Undaria pinnatifida* fucoidan (≥90%, DPGFS2023001), and 100 mg/mL 2′-FL (98%, B08J8DTMGK, Layer Origin, USA) were mixed 1:1 with 25 µg/mL of (GII.4 or GII.17) VLPs and incubated for 1 h at 25°C. Negative control wells (no inhibitor, no Fc-NB26) were subtracted from all OD_450_ values, and untreated VLPs were set as 100% binding. Inhibition (%) was calculated as: 1 – (mean OD₄₅₀ treated / mean OD₄₅₀ reference) × 100. The line graphs show results for GII.4 (**A**) and GII.17 (**B**) VLPs with one A-blood type (blue), two B-blood types (light green and green), and one O-blood type (orange). IC₅₀ values were determined using Excel and indicated by dashed lines. IC₅₀ values were estimates for ulvan, depyrogenated *Fucus vesiculosus* fucoidan, and *Undaria pinnatifida* fucoidan since two measurements above and below 50% inhibition were not available across all replicates, which is typically required (16).

Using an established *in vitro* surrogate neutralization assay (4–6), we evaluated the ability of fucoidan and ulvan extracts (Table 1) to inhibit norovirus virus-like particle (VLP) binding to HBGA-containing human saliva samples. Inhibition profiles were comparable for two clinically significant norovirus genotypes, GII.4 (Fig. 2A) and GII.17 (Fig. 2B), but clear differences in inhibition capacities were observed. Maritech *Fucus vesiculosus* fucoidan demonstrated the most potent and consistent inhibitory activity for both genotypes with the lowest half-maximal inhibitory concentration (IC₅₀) values, where ^GII.4^IC₅₀ = 0.71–3.81 mg/mL and ^GII.17^IC₅₀ = 0.82–1.42 mg/mL for A, B, and O blood types. In contrast, the other extracts showed weaker and more variable inhibition profiles, where ulvan ^GII.4^IC₅₀ = 12.01–48.25 mg/mL and ^GII.17^IC₅₀ = 10.10–44.77 mg/mL; depyrogenated *Fucus vesiculosus* fucoidan ^GII.4^IC₅₀ = 8.46–100.59 mg/mL and ^GII.17^IC₅₀ = 31.50–99.95 mg/mL; and depyrogenated *Undaria pinnatifida* fucoidan ^GII.4^IC₅₀ = 3.47–50.50 mg/mL and ^GII.17^IC₅₀ = 18.60–67.75 mg/mL. The control 2′-FL was also slightly weaker, ^GII.4^IC₅₀ = 15.68–22.28 mg/mL and ^GII.17^IC₅₀ = 32.75–52.46 mg/mL, and was comparable to previous data (4).

**TABLE 1:** Fucoidan and ulvan extracts

	(% w/w)				Monosaccharides (% mol)						
Extract	Total sugars	Uronic acid	Sulfate	Polyphenolics	Fucose	Xylose	Mannose	Galactose	Glucose	Arabinose	Rhamnose
Maritech Fucus vesiculosus fucoidan	74.5	4.1	27.8	5.5	58.4	10.2	7.0	10.6	13.1	0.7	0
Ulvan	39.6	21.0	20.7	0.2	1.1	9.6	1.3	3.4	2.7	0.6	81.3
Depyrogenated Fucus vesiculosus fucoidan	64.6	4.0	29.8	0.2	82.0	9.5	0	8.5	0	0	0
Depyrogenated Undaria pinnatifida fucoidan	54.3	3.4	27.7	0.2	42.4	0.6	2.1	51.7	2.4	0	0.7

**Fig 3:**
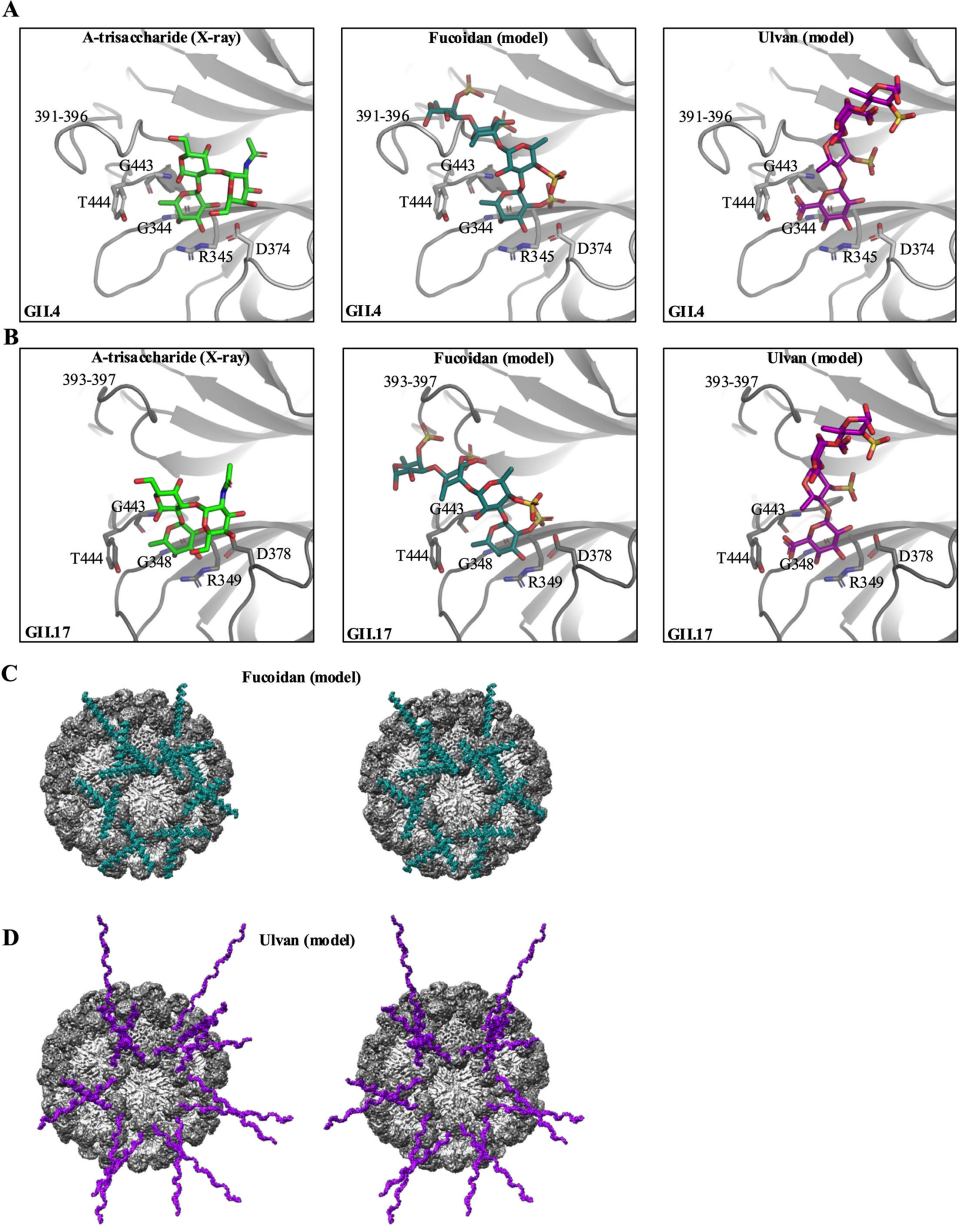
Structural modeling of fucoidan and ulvan bound to the norovirus capsid and possible mode of inhibition. (**A**) GII.4 P domain (light gray) and from left to right: X-ray crystal structure of the GII.4 P domain (light gray) in complex with A-trisaccharide (PDB ID: 4WZT), a model of a four-sugar fucoidan molecule (deep teal), and a model of a four-sugar ulvan molecule (purple) superimposed onto the fucose moiety of the A-trisaccharide (removed for simplicity). The GII.4 P domain residues that bind the fucose of the A-trisaccharide were shown (D374^side-chain^, R345^side-chain^, G344^main-chain^, T444^side-chain^, and G443^main-chain^) and a flexible loop (residues ~391–396) (18–20). The red arrow shows fucoidan near this flexible loop. (**B**) GII.17 P domain (dark gray) and from left to right: the X-ray crystal structure of the GII.17 P domain (dark gray) in complex with A-trisaccharide (5LKC), a model showing a fucoidan molecule (deep teal), and a model showing a ulvan molecule (purple) superimposed onto the fucose moiety of the A-trisaccharide (removed for simplicity). The GII.17 P domain residues that bind the fucose of the A-trisaccharide were shown (D378^side-chain^, R349^side-chain^, G348^side-chain^, T444^side-chain^, and G443^side-chain^) and a flexible loop (residues ~393–397). Models showing stereo views of (**C**) fucoidan and (**D**) ulvan molecules superimposed onto eight dimers on a cryo-EM structure of the GII.17 VLP (EMB-10759). In this model, the fucoidan and ulvan molecules radiate out from the virus particle.

To offer insight into potential molecular mechanisms by which these extracts might inhibit norovirus binding to HBGAs, we first generated three-dimensional (3D) structural models of fucoidan and ulvan with 4- and 64-sugar residues using GLYCAM-Web (17) and subsequently superpositioned into X-ray crystal structures of GII.4 (Fig. 3A) and GII.17 (Fig. 3B) P domains. The four-sugar fucoidan and ulvan were well-accommodated at the HBGA binding pockets on the P domains without any major steric clashes. In the context of the intact particle, a modeled left-handed helical conformation of the 64-sugar fucoidan (Fig. 3C) and ulvan molecules (Fig. 3D) extended outward from the surface of the VLP. This spatial arrangement may contribute to a steric barrier effect, whereby the fucoidan and ulvan molecules might prevent access of HBGA binding, viral aggregation, and/or interference with mobility and orientation during HBGA attachment.

Together, these inhibition data and structural models suggest a dual mode of inhibition—direct competition at the HBGA-binding pocket and a broader steric interference at the virion level. These findings provide a molecular framework for understanding fucoidan and ulvan antiviral action and offer a rationale for further development of fucoidan-based therapeutics targeting norovirus infection, considering ~4 g of fucoidan per day was found to be safe for humans (21, 22).

## References

[1] Hall AJ, Glass RI, Parashar UD. 2016. New insights into the global burden of noroviruses and opportunities for prevention. Expert Rev Vaccines 15:949–951. doi:10.1080/14760584.2016.1178069 27142965 PMC5702916

[2] Harrington PR, Lindesmith L, Yount B, Moe CL, Baric RS. 2002. Binding of Norwalk virus-like particles to ABH histo-blood group antigens is blocked by antisera from infected human volunteers or experimentally vaccinated mice. J Virol 76:12335–12343. doi:10.1128/jvi.76.23.12335-12343.2002 12414974 PMC136916

[3] Ettayebi K, Crawford SE, Murakami K, Broughman JR, Karandikar U, Tenge VR, Neill FH, Blutt SE, Zeng X-L, Qu L, Kou B, Opekun AR, Burrin D, Graham DY, Ramani S, Atmar RL, Estes MK. 2016. Replication of human noroviruses in stem cell–derived human enteroids. Science 353:1387–1393. doi:10.1126/science.aaf5211 27562956 PMC5305121

[4] Rudd PA, Kher G, Tame JRH, Irie H, Haselhorst T, von Itzstein M, Pancera M, Hansman GS. 2024. Human milk oligosaccharide 2’-fucosyllactose guards norovirus histo-blood group antigen co-factor binding site. J Virol 98:e0086524. doi:10.1128/jvi.00865-24 38953656 PMC11264593

[5] Koromyslova A, Tripathi S, Morozov V, Schroten H, Hansman GS. 2017. Human norovirus inhibition by a human milk oligosaccharide. Virology (Auckl) 508:81–89. doi:10.1016/j.virol.2017.04.032 28505592

[6] Weichert S, Koromyslova A, Singh BK, Hansman S, Jennewein S, Schroten H, Hansman GS. 2016. Structural basis for norovirus inhibition by human milk oligosaccharides. J Virol 90:4843–4848. doi:10.1128/JVI.03223-15 26889023 PMC4836343

[7] Schroten H, Hanisch FG, Hansman GS. 2016. Human norovirus interactions with histo-blood group antigens and human milk oligosaccharides. J Virol 90:5855–5859. doi:10.1128/JVI.00317-16 27122582 PMC4907220

[8] Jiang X, Huang P, Zhong W, Tan M, Farkas T, Morrow AL, Newburg DS, Ruiz-Palacios GM, Pickering LK. 2004. Human milk contains elements that block binding of noroviruses to human histo-blood group antigens in saliva. J Infect Dis 190:1850–1859. doi:10.1086/425159 15499543

[9] Shang J, Piskarev VE, Xia M, Huang P, Jiang X, Likhosherstov LM, Novikova OS, Newburg DS, Ratner DM. 2013. Identifying human milk glycans that inhibit norovirus binding using surface plasmon resonance. Glycobiology 23:1491–1498. doi:10.1093/glycob/cwt077 24026239 PMC3816630

[10] Etzold S, Bode L. 2014. Glycan-dependent viral infection in infants and the role of human milk oligosaccharides. Curr Opin Virol 7:101–107. doi:10.1016/j.coviro.2014.06.005 25047751

[11] Tan MTH, Li Y, Eshaghi Gorji M, Gong Z, Li D. 2021. Fucoidan but not 2'-fucosyllactose inhibits human norovirus replication in zebrafish larvae. Viruses 13:461. doi:10.3390/v13030461 33799811 PMC8001738

[12] Hanisch FG, Aydogan C, Schroten H. 2021. Fucoidan and derived oligo-fucoses: structural features with relevance in competitive inhibition of gastrointestinal norovirus binding. Mar Drugs 19:591. doi:10.3390/md19110591 34822462 PMC8617971

[13] Kim H, Lim CY, Lee DB, Seok JH, Kim KH, Chung MS. 2020. Inhibitory effects of Laminaria japonica fucoidans against noroviruses. Viruses 12:997. doi:10.3390/v12090997 32906822 PMC7552056

[14] Hanisch FG, Aydogan C. 2020. Oligosaccharides and viral infection: human milk oligosaccharides versus algal fucan-type polysaccharides. Nestle Nutr Inst Workshop Ser 94:124–132. doi:10.1159/000505338 32176880

[15] Aguilar-Briseño JA, Cruz-Suarez LE, Sassi J-F, Ricque-Marie D, Zapata-Benavides P, Mendoza-Gamboa E, Rodríguez-Padilla C, Trejo-Avila LM. 2015. Sulphated polysaccharides from Ulva clathrata and Cladosiphon okamuranus seaweeds both inhibit viral attachment/entry and cell-cell fusion, in NDV infection. Mar Drugs 13:697–712. doi:10.3390/md13020697 25629385 PMC4344596

[16] Sebaugh JL. 2011. Guidelines for accurate EC50/IC50 estimation. Pharm Stat 10:128–134. doi:10.1002/pst.426 22328315

[17] Grant OC, Wentworth D, Holmes SG, Kandel R, Sehnal D, Wang X, Xiao Y, Sheppard P, Grelsson T, Coulter A, Miller G, Foley BL, Woods RJ. 2025. Generating 3D models of carbohydrates with GLYCAM-Web. bioRxiv:2025.05.08.652828. doi:10.1101/2025.05.08.652828 41872371

[18] Hansman GS, Kher G, Svirina AD, Tame JRH, Hartley-Tassell L, Irie H, Haselhorst T, von Itzstein M, Rudd PA, Pancera M. 2024. Development of a broad-spectrum therapeutic Fc-nanobody for human noroviruses. J Virol 98:e0070724. doi:10.1128/jvi.00707-24 38953655 PMC11264634

[19] Singh BK, Leuthold MM, Hansman GS. 2015. Human noroviruses’ fondness for histo-blood group antigens. J Virol 89:2024–2040. doi:10.1128/JVI.02968-14 25428879 PMC4338890

[20] Singh BK, Koromyslova A, Hefele L, Gürth C, Hansman GS. 2015. Structural evolution of the emerging 2014-2015 GII.17 noroviruses. J Virol 90:2710–2715. doi:10.1128/JVI.03119-15 26699640 PMC4810701

[21] Abe S, Hiramatsu K, Ichikawa O, Kawamoto H, Kasagi T, Miki Y, Kimura T, Ikeda T. 2013. Safety evaluation of excessive ingestion of mozuku fucoidan in human. J Food Sci 78:T648–51. doi:10.1111/j.1750-3841.2012.02966.x 23465035

[22] Citkowska A, Szekalska M, Winnicka K. 2019. Possibilities of fucoidan utilization in the development of pharmaceutical dosage forms. Mar Drugs 17:458. doi:10.3390/md17080458 31387230 PMC6722496

